# Mesh migration following abdominal hernia repair: A case report, and literature review

**DOI:** 10.22088/cjim.13.4.815

**Published:** 2022

**Authors:** Ali Jangjoo, Mohammad Ebrahim Kalantari, Tooraj Zandbaf

**Affiliations:** 1 Department of General Surgery, Surgical Oncology Research Center, Mashhad University of Medical Sciences, Mashhad, Iran; 2 Minimally Invasive and Bariatric Surgeon, Surgical Oncology Research Center, Mashhad University of Medical Sciences, Mashhad, Iran; 3 Department of General Surgery, Faculty of Medicine, Mashhad Medical Sciences, Islamic Azad University, Mashhad, Iran

**Keywords:** Surgical mesh, Hernia repair, Mesh migration. Hernioplasty complication, Incisional hernia

## Abstract

**Background:**

Postoperative hematoma and seroma, foreign body reaction, infection, mesh rejection, and fistula formation are the complications associated with the use of surgical mesh. Mesh migration is a rare but serious and challenging complication after hernia repair. When this happens, infection, abscess, fistula, and bowel obstruction are the most common sequelae.

**Case presentation:**

Our patient was a 62-year-old woman with a history of appendectomy 30 years ago and then underwent 3 incisional hernia repair surgeries which the last one was 5 years ago using laparoscopic IPOM. The patient was nominated for surgery with a diagnosis of recurrent incisional hernia. The patient underwent laparotomy and after enterolysis, a small bowel loop was seen that adhered to McBurney's region, which was released. There was a mass inside the small bowel. Resection and anastomosis of the involved intestine were performed. After enterotomy, it was determined that this mass was the mesh used in the previous surgery.

**Conclusion:**

Mesh migration is a rare consequence of incisional hernia repair with a prosthetic mesh. It can happen years after a hernia repair and it is additionally crucial to consider as a differential diagnosis in all patients who show unusual symptoms or abdominal pain.

The most common complication of abdominal surgery is an incisional hernia, which affects approximately 10-15% of patients. The recurrence rate after the anatomic repair is almost 20-45 %. Prosthetic surgery has dramatically reduced the recurrence rate from 50% to 10%–20% (1, 2). Hernia repair is one of the foremost common surgical methods performed universally. It is estimated that there are over 20 million hernia repair surgeries each year around the world (3). Using surgical meshes has become the hernia repair method of choice. It has demonstrated to have a lower rate of recurrence. There are currently around 70 different types of meshes available in the market. The surgical mesh securely strengthens the weaker area and enables tension-free healing, allowing fibro collagenous tissue to be incorporated more easily (3, 4). We report an unusual case of transmural migration of composite mesh to the small intestine five years after incisional hernia surgery, which manifested as chronic abdominal pain. Our work has been documented by the SCARE guidelines. 

## Case presentation

The patient was a 62-year-old woman referred to the General Surgery Department due to the protrusion and bulging on the appendectomy incision. She had a history of appendectomy 30 years ago and then underwent 3 incisional hernia repair surgeries in which the last one was 5 years ago using laparoscopic IPOM.

**Figure 1.**
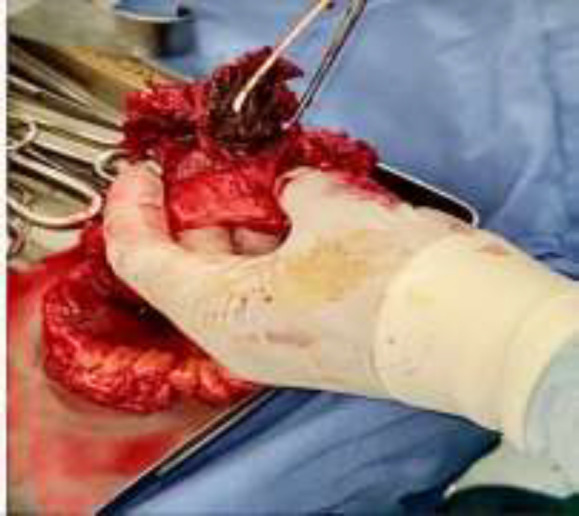
Evidence of the mesh completely intraluminal

Over the years, the patient has had no symptoms and signs of bowel obstruction and has only complained of chronic intermittent abdominal pain. On physical examination, there was only one bulging on the McBurney incision. All lab tests were unremarkable. CT scan of the abdomen and pelvis confirmed incisional hernia. The patient was nominated for surgery and underwent laparotomy after general anesthesia. After enterolysis and intestinal adhesions removal, a small bowel loop that adhered to McBurney's region was released. There was a mass inside the small intestine. In the surrounding area, there was no sign of an abscess, fistula, or sinus formation. Resection and anastomosis of the involved intestine were performed. After enterotomy, it was determined that this mass was the mesh used in the previous surgery (fig. 1). The patient was discharged from the hospital 5 days after surgery, in good general condition and without any particular problems.

## Discussion

While the incidence of recurrence of incisional hernias after the simple sutured repair is more than 60%, applying mesh reduced the recurrence rate to around 30%. Mesh repair is especially crucial for incisional hernias more than 4 cm in diameter because the risk of bigger hernia recurrence is likely related to the tension imposed on the repair (2).

Theodor Billroth proposed in 1890 that using a prosthetic material to close the hernia defect was the best technique to repair hernias. Surgeons now believe that using a prosthetic mesh to repair hernias is the best option. Composite meshes are divided into two types: absorbable and permanent (non-absorbable). Absorbable composite meshes which must be hydrated before use, are not modifiable and can affect the success of tissue ingrowth which is ideal to minimize probable complications. Permanent composite meshes can be customized to fit a variety of applications and have less visceral adhesions and problems. Even though surgical meshes reduce recurrence rates, they have side effects such as infection, adhesion, and bowel obstruction (3).

The cause of mesh migration is not yet known. A primary mechanical migration and a secondary migration are two separate mechanisms that have been proposed. One reason for mesh migration in adjacent anatomical areas might be an insufficient fixation on the fascia or improper stabilization by external forces. Later on, the mesh may dissolve into the surrounding tissue. Secondary migration may also occur through trans-anatomical planes as a result of the foreign body reaction (1,5). Another idea proposes that the presence of adhesions from earlier hernia repair may predispose to new adhesions which make migration happen. Some researchers believe migration occurs when a piece of the bowel is mistakenly included in the mesh's fixation. The mesh's cut edges may irritate the surface of nearby organs, triggering an inflammatory reaction that leads to weakening and erosion (1, 6). It seems that the history of multiple surgeries and the presence of severe adhesions have been effective in mesh migration in our patient.

The first case of mesh migration after incisional hernia surgery was described by Herrera et al. Both composite and no absorbable mesh migrations have been reported in the literature, but it is invariably linked with clinical signs and symptoms such as abscess formation, enterocutaneous fistula formation, or intestinal obstruction (4). The unique feature of our patient was that she did not have any complications such as abscess or fistula formation and intestinal obstruction.

Not only complications following mesh contamination depend on traits of mesh-like material, pore size, filament structure, and coating, but also a surgical method like the plane of mesh placement, sort of fixation, tissue handling must be considered. Patient bad dietary habits and concurrent enterotomy along with mesh used during hernia repair also can accelerate the mesh contamination possibility (3, 7).

Suture fixation is 93% less likely to have complications, but this is not statistically significant. It has been advised that suture fixation might also additionally increase the hazard of post-operative pain, wound contamination, and operating time. Also, regular utilization of absorbable tack quotes decreased post-operative pain and seroma formation (8). One of the factors that cause this complication is the use of a large mesh graft (20×15 cm) for a minor defect, especially if it is not properly applied. This leaves a gap between the graft and the abdominal wall, causing intestinal entrapment (6).

Radiologists, who are usually concerned at the beginning and throughout a patient's hospital stay, ought to be suspicious of findings suggesting migrated mesh. Surgical staples in unusual locations as well as abnormal configurations ought to be investigated immediately via CT to gauge for mesh-related pathology, and notably intra-luminal migration. Many synthetic and biologic meshes are difficult to see on CT. Therefore, secondary findings of mesh migration such as mural inflammation, abnormal bowel course or contour, an unusual appearance of bowel contents, as well as, ectopic staples should prompt radiologists to consider intraluminal migration (9). Although mesh migration causes a potentially fatal long-term complication, the mesh migration rate is significantly lower than the recurrence rate after a non-mesh hernia repair. There is no better alternative for treating incisional and abdominal hernias than mesh placement (10). It seems that choosing the appropriate size and completely fixing the mesh can greatly prevent this complication. However, more studies are needed to fully determine the nature of the migration mesh.

In conclusion mesh migration is a rare consequence of incisional hernia repair with a prosthetic mesh. It can happen years after a hernia repair and it is additionally crucial to consider as a differential diagnosis in all patients who show unusual symptoms or abdominal pain. The results of mesh migration are serious and may require surgical intervention. For these reasons, we suggest surgeons include mesh migration and its potential complications during the consent process.
